# Author Index for Volume 104

**DOI:** 10.1038/bjc.2011.211

**Published:** 2011-06-07

Author Index for Volume 104 Aalfs, CM 1356 Abadie, C 1517 Abbotts III, R 653 Abdah-Bortnyak, R 1649 Abenhardt, W 1071 Abernethy, AP 390 Abnet, CC 1511 Abud, L 1854 Acerini, CL 746 Acheson, N 1836 Adlard, J 1356 Aglietta, M 1686 Ahlbom, A 228 Ahmad, I 664 Ahmedzai, SH 1551 Ahn, JS 559 Ahson, G 1836 Aigelsreiter, A 1641 Aird, KM 1575 Aittomäki, K 1356 Akino, T 819 Akiyama, K 819 Akslen, LA 921 Al Ashari, M 1362 Alain, T 290 Alanko, T 599 Albert, A 989 Aldrich, A 1575 Alessi, DR 1116 Alexandre, J 1670 Ali, AMG 564, 693 Alibhai, S 1377 Al-Khyatt, W 1393 Allen, M 1675 Allen, NE 1487, 1493 Allgood, PC 1680 Al-Mahmood, S 496 Alman, BA 1452 Alonso, C 1106 Alonso, R 1356 Alsiary, R 1602 Altieri, DC 629 Amagasa, T 850 Amant, F 863 Amatschek, S 469 Amiano, P 1493 Amir, E 1377 Amira-Bouhidel, F 1739 Amodio, E 433 Ancona, E 427 Anders, M 1426 Andersen, PK 520 Anderson, J 175 Anderson, KS 1896 Anderson, R 1836 Andersson, S 110 Andreeff, M 91 Andrews, D 1365 Andrikopoulos, P 1151 Angeloni, C 248 Antoine, M 1739 Antoniou, AC 1356 Aoki, M 714 Ardizzoni, A 1372, 1929 Arigami, T 714 Arkenau, H-T 392 Armenoult, LSC 971 Armesilla, AL 1564 Arner, P 441 Ashcroft, M 1151 Ashdown-Barr, L 915 Ashley, S 1675 Ashton, KM 790 Asou, H 91 Atula, T 1890 Au, JSK 208 Au, TCC 1000 Auer, G 110 Ausems, MGEM 1356 Austin, J 915 Avis, M 255 Avruch, J 24 Azzariti, A 769

Baba, H 300 Baccarini, M 229, 1240 Bachleitner-Hofmann, T 543 Bae, K-s 605 Baert-Desurmont, S 1517 Baeten, CG 37 Baglietto, L 524, 693 Bagni, B 1079 Bahl, A 1587 Bailey, D 1836 Baird, R 43 Bajetta, E 1256 Baker, JB 488 Balkwill, A 1487 Balmer, SM 726 Bandiera, E 1418 Banfield, A 587 Bao, Y 441 Bapat, B 1906 Baracos, VE 1288 Barbachano, Y 43 Barbanti, G 613 Barbany-Bustinza, G 1356 Barber, B 1848 Bardeesy, N 24 Bargagli, G 613 Barod, R 1151 Barricarte, A 1493 Barriuso, J 226 Barsky, SH 1628 Bassano, C 1372, 1929 Bast, A 437 Basu, B 1 Bates, TE 193 Batra, SK 1038 Bauernhofer, T 1641 Beard, R 620 Bearzi, I 1786 Beaudin, J 989 Becker, S 110 Beesley, J 1356 Bell, SM 1602 Bellanova, C 248 Bencko, V 1797 Benetou, V 1493 Bengtsson, N-O 899 Benítez, J 1356 Bennett, RL 571 Bennouna, J 413 Benoy, IH 1472 Beral, V 4, 1487 Berditchevski, F 1611 Bergamelli, S 1418 Bergmann, M 543 Berkhof, J 685 Bernardini, S 1770 Bernatsky, S 1478 Bernhard, H 948 Bernoud, R 1739 Berntsson, SG 1747 Bertheau, P 1739 Berthier-Vergnes, O 155 Bertolini, F 1079 Bethe, U 1691 Betts, G 1168, 1319 Bex, A 741 Beyene, J 1377 Bhandari, S 1236 Bhattacharyya, T 1151 Bieche, I 272 Bienkowski, M 968 Biernacka, KM 1587 Bignotti, E 1418 Bijnsdorp, IV 1185 Billan, S 1649 Billiot, F 1144 Bing, C 441 Biondo, A 43 Birch-Machin, M 1319 Birgisson, H 1619 Biscotti, T 1786 Bjørge, T 921 Blair, C 532 Blakely, J 68 Blann, A 1505 Blann, AD 1822 Blay, J-Y 1544 Blighe, K 1342 Blows, FM 693 Bocci, G 769, 1262 Böckelman, C 1890 Bocquet, C 1739 Boddy, AV 593, 1869 Bodin, I 110 Boeing, H 1493 Boffetta, P 1493, 1797 Boivin, M 989 Bonanni, B 1356 Bond, J 1602 Bonfiglio, TA 353 Bongaerts, M 496 Bonnier, P 332 Bono, P 599, 1686 Booth, CJ 1401 Borden, EC 957 Borgen, E 1434 Borriello, M 1716 Borthwick, K 324 Borup, R 830 Bose, D 1270 Boselli, S 1686 Bosma, AJ 1913 Bougeard, M 1517 Boult, JKR 83 Bourstyn, E 1739 Boutron-Ruault, M-C 1493 Bouville, A 181 Bowen, RL 120 Boye, K 1434 Boykin, C 781 Boyle, RW 1106 Bozic, B 1246 Bozzetti, C 1372, 1929 Bracarda, S 1256 Brandone, J-M 332 Brandt, R 635 Bray, F 178 Breene, RAL 746 Brennan, MF 1840 Brennan, P 1797 Brenner, AV 181 Brenner, H 1779 Brenton, JD 361 Brewer, C 1356 Brewster, DH 60, 1791 Bright, JJ 448 Brinkman, M 524 Broderick, P 1049 Broeders, MJM 910 Brotherton, JML 886 Brouste, V 1544 Brown, H 1319 Brown, J 1342 Brown, JM 1805 Brown, R 1313 Brown, S 1564 Browne, PV 281 Browne, S 1697 Bruchim, R 1049 Brudler, O 1071 Brugger, W 1071 Brugnara, S 1816 Brummendorf, TH 1691 Brunet, J 1356 Brüning-Richardson, A 1602 Bryant, HE 1098 Buchanan, DD 1906 Büchner, FL 1493 Buckland, G 1493 Bueno-de-Mesquita, HB 1493 Buffa, FM 1168 Bui, B 1544 Bukholm, I 1434 Burdach, S 948 Burgers, JA 1325 Burke, W 750 Burns, P 1602 Burton, H 1656 Busch, DH 948 Butini, S 281 Buxton, ILO 1628 Byers, RJ 514 Byrne, C 587 Byrnes, GB 903 Bytheway, I 635 Bzhalava, D 178

Cadman, L 915 Cadron, I 863 Cahen-Doidy, L 1739 Cai, C 1319 Caiazza, F 338 Cailleux, A-F 1517 Cairns, BJ 4 Cairns, DA 1602 Caldas, C 693 Caldon, LJM 1551 Califano, R 750 Caligo, MA 1356 Calvert, H 593 Calza, S 1418 Cameron, D 1529 Camilloni, L 248 Camisa, R 1372, 1929 Campbell, FC 1007 Campbell, H 1529 Campbell, NC 1697 Campiani, G 281 Camps, C 1168 Canovas, D 1098 Canu, B 1262 Capellá, G 735, 1519 Capelle, L 1854 Capelli, S 1372 Capparucci, P 248 Capriotti, F 1185 Caradec, J 1056 Card, T 193 Carmichael, J 750 Carozzi, F 248 Carpenter, LM 1227 Carpenter, R 120 Carstensen, J 899 Carter, R 353 Cartwright, JE 83 Carvajal-Carmona, L 369 Casali, PG 1686 Cascinu, S 1786 Cassidy, J 1564 Cavalli, S 1929 Cesario, A 1877 Chakraborty, S 448, 1038 Challen, C 1319 Chan, ATC 1000 Chan, CML 1000 Chan, KW 841 Chan, S 407 Chan, YP 841 Chang, H-M 605 Chang, MS 166 Charles, KA 1288 Chau, I 43 Chau-in, S 1313 Chen, EX 756 Chen, X-C 1356 Chen, Y 308, 892, 1013 Chen, Y-H 781 Chenevix-Trench, G 1356 Cheng, CWS 941 Cheng, J 1401 Cheng, KK 369 Cheng, T 1410, 1931 Cheriyath, V 957 Cherny, SS 369 Chester, JD 75 Cheung, AHK 1000 Cheung, KL 1393 Cheung, KT 790 Cheung, MT 1000 Chhaya, V 43 Chia, KS 871 Chia, VM 763 Chiarappa, P 769 Chiarion-Sileni, V 1816 Chie, W-C 587 Chiras, J 1854 Chiriacò, G 613 Chiyomaru, T 376, 808 Cho, SJ 1126 Choi, IJ 1126 Choi, MS 559 Choi, S-c 369 Choi, SH 1027 Choi, SY 1730 Choi, Y 1730 Choi, YJ 166 Chong, TW 941 Chow, W-H 537, 1511, 1797 Chowdhury, S 407, 741, 1656 Christensen, K 520 Christensen, S 419 Christison, J 915 Chua, W 1288 Chuang, S-C 1178 Chuang, TH 1410, 1931 Chung, CC 369 Chung, IY 1730 Ciardiello, F 427 Clark, DI 60 Clark, EA 488 Clark, SE 120 Clark, SK 1236 Clarke, AE 1478 Clarke, SJ 1288 Clavel-Chapelon, F 1493 Coco, P 1686 Coghill, AE 763 Coit, DG 1840 Colditz, GA 882 Coleman, RE 1665 Colin, S 496 Collins, KA 1551 Collins, SI 1500 Colucci, G 1079, 1816 Comamala, M 989 Conca, R 613 Confortini, M 248 Congedo, MT 1877 Conlon, S 480 Connor, T 1151 Conrad, H 948 Constantinidou, A 1675 Conte, PF 1079 Conway, EL 886 Cook, M 1356 Cooke, SL 361 Coombes, RC 1342 Cooper, PA 75 Corso, G 1770 Costalat, R 1854 Cottet, V 1493 Cotton, S 255 Couch, FJ 1356 Courtney, R 1862 Couto, E 1493 Crafa, P 1372, 1929 Crawford, RAF 361 Cremolini, C 1262 Crespi, CM 228 Cretella, M 1770 Cronin-Fenton, DP 188, 1804 Crowe, F 1493 Cruickshank, M 255 Cubie, HA 1221 Cui, T 1013 Cullis, ER 83 Cummings, J 719 Cunningham, D 43 Curran, WJ 1365 Currie, MJ 51 Currow, DC 390 Curzon, B 1356 Cuschieri, K 1221 Cvitkovic, E 272

Dabelsteen, S 830 Dachs, GU 51 D’Addetta, A 248 Daduang, J 1313 Daemen, A 863 Daftary, D 1906 Dahiya, R 308, 892 Dahm, CC 1493 Dai, W 1313 Dal Maso, L 1207 D’Alessio, G 1716 Dallas, NA 1270 Dalton, SO 188, 1804 Damery, S 927 Danesi, R 1262 Darbyshire, J 1529 Darcy, KM 353 Darling, JL 1564 Darmanis, S 1619 Das, N 1836 Davidson, R 1356 Davies, JM 1067 Davis, M 875 Dawson, S-J 693 Day, FL 265 de Bazelaire, C 1739 de Bono, JS 1 De Braud, F 1686 de Gelder, R 1214 de Jong, S 1278 de Koning, HJ 1214 de la Cour, ZD 1202 de la Fouchardière, A 155 De Lorenzo, C 1716 De Marco, G 1854 de Mol, BAJM 1325 De Moor, B 863 De Pas, T 1686 de Roquancourt, A 1739 De Rubertis, G 613 De Salvo, GL 1816 de Thé, H 1739 De Vita, F 427 de Vries, EG 1278 Dearnaley, D 175 Decarli, A 1207 Dekker, E 37 Dekker, LV 653 Del Bianco, P 1816 Del Bufalo, D 769 Del Genio, A 427 Del Giovane, C 1079 Del Tacca, M 769 Del Vecchio Blanco, G 1770 Delacotte, N 1056 Dell’Aniello, S 1558 den Heeten, GJ 910 Deng, G 892 Dent, T 1656 Depenni, R 1079 Dernede, U 407 Despierre, E 863 Devi, GR 1575 Devriese, LA 1913 Dewar, J 1246 Di Cecilia, S 1770 Di Desidero, T 1262 Di Iulio, J 265 Di Maio, M 427 Di Martino, N 427 Di Paolo, A 1262 Di Pierro, C 248 Diamond, T 1007 Dicker, AP 1365 Dicks, E 1906 Dickson, MA 1862 Dieras, V 1670 diGioia, D 1071 Dillner, J 178 Dimitropoulou, P 875 Dirix, LY 1472 Dite, GS 903 Dive, C 719 Doki, Y 707 Dolled-Filhart, M 68 Domchek, S 1384 Domchek, SM 1356 Donaghy, M 1221 Donders, R 910 Dong, Y 893 Donne, AJ 324 Donovan, JL 875 Douglas, F 1356 Downing, S 1356 Dowswell, G 927 Dowty, JG 903 Draisma, G 1214 Draper, G 228 Dredge, K 635 Drozdovitch, VV 181 D’Souza, G 1896 Duanmu, J 1401 Duce, SL 1116 Duffy, SW 120, 871, 1656, 1680 Duhamel, A 1544 Duiker, EW 1278 Dutta, D 488 Dvrokind, I 1649 Dziunycz, P 1106

Easton, D 175 Easton, DF 1356, 1656 Eatock, M 593 Eccles, D 1356 Edwards, DR 664 Edwards, J 1920 Edwards, R 915 Eeles, RA 175, 578, 1356, 1656 Eger, A 469 Ehrencrona, H 1356 Eisen, A 1384 Eisenhauer, EA 756 El-Bahrawy, MA 1313 Elder, DJE 1755 Ellis, IO 693, 1393 Ellis, LM 1270 Elsir, T 1747 El-Tanani, M 1007 Endo, Y 798 Engeset, D 1493 English, DR 524 Enokida, H 376, 808 Erichsen, R 1202 Erlander, MG 1762 Escudier, B 1144 Espié, M 1739 Esteban, MA 1151 Evans, DG 578, 1356 Evans, P 281 Evans, T 1505 Everard, J 1665 Eveson, JW 1459 Evseenko, VV 181

Facchetti, F 1418 Faivre, S 272 Falchetti, M 1418 Falcone, A 1262, 1786 Fallowfield, L 1535 Faloppi, L 1786 Fan, F 1270 Fan, X-E 781 Farace, F 1144 Farella, A 427 Farewell, D 1535 Farewell, V 1535 Farkas, DK 1202 Farrugia, D 1067 Faulstich, F 1334 Fearon, KCH 441 Federici, G 1770 Feldman, D 1049 Feliu, J 226 Feliubadaló, L 1356 Ferguson, M 1877 Ferrari, P 1493 Ferraroni, M 1207 Ferro, V 635 Fiaschi, AI 613 Fields, RC 1840 Figueiredo, C 198 Fina, F 332, 1364 Fiocchi, F 1079 Fioravanti, A 769, 1262 Fiorentino, F 1085 Fischer, PM 653 Fisher, AL 460 Fitamant, J 24 Flandre, E 1739 Flatmark, K 1434 Fleming, J 664 Fleming, S 1116 Flint-Richter, P 1049 Fodstad, Ø 1434 Foley, K 390 Fombon, IS 1564 Fontana, A 1079 Ford, E 1535 Formosa, A 1770 Fornander, T 899, 1762 Forster, JA 1135 Forsyth, PA 290 Fort, E 1836 Fortuzzi, S 1356 Foulis, AK 726 Foulkes, WD 1384, 1478 Franceschi, S 1207 Francini, E 613 Francini, G 613 Frater, A 915 Frebourg, T 1517 Fredericksen, Z 1356 Freier, W 1071 Friedland, PL 1246 Friedrich, RE 138 Friend, E 587 Friis, S 188, 1804 Friis-Hansen, L 830 Frost, D 1356 Frost, K 1303 Frumento, P 1262 Frydenberg, M 934, 1930 Fujiwara, K 353 Fujiwara, Y 707 Fukuhara, H 1349 Fukushima, M 1185 Fung, SS 369 Furukawa, M 1160 Furuyama, N 798

Galizia, E 1786 Galizia, G 427 Gallinger, S 1906 Gambier, N 1238 Ganesan, R 1505, 1611 Gao, D 441 Gao, F 871 Gao, W 1704 Gao, Y-T 1511 Garant, KA 290 Garantziotis, S 542 Garcia-Barceló, M-M 369 García-Martínez, JM 1116 Gardini, G 1372, 1929 Garlick, DS 629 Garnæs, E 830 Gasparro, D 1372, 1929 Gattens, M 746 Gatter, KC 1877 Gaur, P 1270 Gee, HE 1168 Geisler, J 1059 Gelderblom, H 37 Georgoulias, V 316 Gershman, S 1384 Gerster, M 520 Gerunda, GE 1079 Ghadirian, P 1384 Ghaneh, P 1440 Giacchetti, S 1739 Giampieri, R 1786 Gibson, W 1575 Gilbert, D 407 Gilbert, FJ 578 Gilbert, R 875 Giles, GG 524, 903 Gillatt, D 1587 Gillet, P 1238 Giorgi Rossi, P 248 Gitsch, G 1071 Glabbeke, MV 1544 Glimelius, B 1619 Godwin, A 1356 Goedert, JJ 433 Goel, HL 629 Goggins, M 1296 Gök, M 685 Golby, S 1836 Goldenring, JR 33 Goldwasser, F 1670 Gonda, TJ 635 Gönen, M 1840 Gonzalez, CA 1493 Gonzalez-Perez, RR 128 Goodman, KA 1840 Goodyer, M 281 Gordon, C 1478 Gordon, J 1836 Gordon, N 1611 Gore, ME 399, 750 Gornall, R 1611 Goto, Y 464 Grabar, S 1670 Grasl-Kraupp, B 1303 Gratus, C 927 Graubard, BI 433 Gravells, P 1098 Gray, NM 255, 1697 Grazzini, G 248 Green, AR 1393 Green, J 1487 Green, JA 1521 Green, RC 1906 Green, S 68 Greenberg, D 564, 1810 Greenfield, S 927 Gregersen, H 1202 Grelier, G 387 Greystoke, A 719, 750 Griesbacher, A 1641 Grieve, R 927 Griffin, M 593 Griffioen, AW 1185 Grignagni, G 1686 Grimm, C 223 Gromiec, J 1797 Groselj-Strele, A 1641 Gross-Goupil, M 1144 Grosse-Kracht, S 469 Gruber, SB 735, 1519 Grulich, A 886 Grusch, M 1303 Gualberto, A 68 Guichard, S 1116 Guida, M 1816 Guigay, J 1691 Guilfoyle, MR 1810 Guillén-Ponce, C 224 Guillevin, R 1854 Guilmain, W 496 Guinó, E 735 Gulliford, M 1704 Guo, S 128 Guttery, DS 1342 Guy, M 175 Gwak, G-Y 559

Habas, C 1854 Haddad, R 1896 Haenen, GRMM 437 Hagihara, J 228 Haglund, C 1890 Hagström, J 1890 Hague, A 1459 Haick, H 1649 Hakim, M 1649 Hall, AL 175 Hall, GD 1602 Hall, JS 971 Hall, SJ 1697 Hallmans, G 1493 Hamada, J-I 798 Hambleton, J 413 Hamdy, FC 875 Hamilton, P 1007 Hamilton, R 1529 Hamilton, W 934, 1930 Hammond, E 635 Hampson, IN 324 Hampson, L 324 Han, C 1822 Hancock, BW 1665 Handley, P 635 Handra-Luca, A 1296 Hank Juo, S-H 1178 Hansen, RP 934, 1249, 1930 Harao, M 300 Harbison, CT 488 Hareyama, M 1724 Hårklau, L 1434 Harnden, P 1135 Harris, AL 1168, 1822 Harris, F 1810 Harriss, D 175 Hartle, J 746 Hartmann, C 1747 Hartschuh, W 1334 Harvey, BJ 338 Hatae, M 353 Hatch, M 181 Hatschek, T 899 Haug, U 1779 Hauler, F 1106 Hauptmann, M 1913 Hava, N 1342 Haward, R 1529 Hayashi, Y 798 Hayden, P 281 Hayes, DN 545 Haynes, K 1810 Häyry, V 1890 Hayward, N 1067 He, X 235, 324 Healey, S 1356 Heer, R 1869 Heijnsdijk, EAM 1214 Heinemann, V 1071 Helgason, HH 1913 Hellman, K 110 Hellman, U 110 Hellström, A-C 110 Henrich, H 345 Heo, JS 1027 Hetzel, MR 1755 Hickson, ID 653 Hida, K 819 Hida, Y 819 Higashino, F 819 Higginson, IJ 1704 Hill, ADK 338 Hill, C 1144 Hinch, E 1055 Hinkal, GW 387 Hinoda, Y 308 Hinz, A 345 Hiraike, H 1349 Hirata, H 308 Hirata, S 300 Hirte, HW 756 Hixon, ML 68 Ho, JW 369 Ho, S 369 Hoang-Xuan, K 1854 Hocking, JS 886 Hodge, A 524 Hofbauer, GF 1106 Hogervorst, FB 1356 Hoh, L 1098 Hohenberger, P 1544 Holland, R 910 Hollenbeck, AR 537 Holly, JMP 1587 Holmlund, B 1762 Holt, S 1551 Homer, J 1168 Hong, H 1452 Hong, S-M 1296 Hong, YS 605 Hopper, JL 524, 903 Horgan, PG 726 Horikawa, T 1160 Horne, LS 460 Hosking, FJ 1049 Hospers, GAP 1193 Hotte, SJ 756 Hou, M-F 1178 Houlston, RS 369, 1049 Hruban, R 1296 Hsieh, C-C 629 Hsu, SM 982 Hu, Z 1401 Huang, PH 982 Huang, S 235 Huber, O 1013 Hughes, A 719 Hughes, G 1836 Hui, EP 1000 Hui, SM 369 Hui, TC 369 Huiart, L 1558 Hundsberger, H 469 Hundt, S 1779 Huntsman, D 693 Hurtz, HJ 1071 Hurwitz, H 413 Hussaini, HM 460 Hutson, R 1602 Hutson, T 741 Hutton, J 578 Huynh, H 941

Ikeda, Y 1349 Ikushima, H 241 Ikuta, Y 300 Illidge, TM 719 Ilson, DH 1840 Im, Y-H 559 Imai, K 300 Imamura, T 1882 Inaba, T 91 Inenaga, S 1882 Ingram, SM 1575 Innocenti, R 427 Inoue, M 300 Iossa, A 248 Irie, A 300 Irlam, JJ 971 Isaacs, C 1356 Ishigami, S 714 Ishizu, A 241 Ismail-Khan, R 1828 Isoniemi, H 599 Iurisci, I 1670 Izatt, L 1356 Izumi, H 1882 Izumi, K 505

Jacobs, H 437 Jacobsen, HJ 1434 Jacques, N 1144 Jager, A 1356 Jain, M 1038 Jakes, RW 871 Jalava, T 1686 Jang, G 605 Jang, K-T 1027 Janin, A 1739 Janout, V 1797 Jaskolski, DJ 968 Javot, L 1238 Jearanaikoon, P 1313 Jefferies, SJ 1810 Jeffery, C 1810 Jefford, M 265 Jenab, M 1493 Jenkins, MA 903 Jenkins, V 1535 Jensen, AB 419 Jerevall, P-L 1762 Jernström, H 1356 Ji, B-T 1511 Jimenez-Linan, M 361 Jin, L 91 Jirström, K 1619 Joensuu, H 1686 Johannessen, H-O 1434 Johannsson, OT 1356 Johansson, C 1619 Johansson, I 1493 Johnson, CD 587 Johnson, N 1836 Johnston, M 1697 Johnston, PG 480 Johnston, RN 290 Johnston, S 1675 Johnston, SJ 1393 Johnstone, AP 83 Jonat, W 1071 Jones, J 927 Jones, JL 120 Jones, L 407 Jones, R 741 Jubb, AM 1877 Judson, I 1544 Jung, J 166

Kaaks, R 1493 Kai, M 1724 Kaizaki, Y 1160 Kaklamanis, L 316 Kakuguchi, W 819 Kalaycio, ME 957 Kalimutho, M 1770 Kalland, KH 921 Kamangar, F 1511 Kamdar, RP 1724 Kamishima, T 241 Kanakasabai, S 448 Kanayama, H 241, 505 Kang, BH 629 Kang, E 1730 Kang, ES 559 Kang, Y-K 605 Kannappan, V 1564 Kaplan, R 1529 Kappeler, C 1686 Karami, S 1797 Karp, DD 68 Karpathakis, A 43 Karran, P 1869 Kataoka, K 593 Kato, S 1349 Kauderer, J 353 Kawahara, K 376 Kawakami, K 376, 808 Kay, EW 480 Kearins, O 1680 Keates-Porter, S 1836 Keating, PJ 790 Kefford, R 392 Keilholz, U 1691 Keller, E 664 Kelsen, DP 1840 Kelsey, KT 1896 Kennedy, H 1393 Kerber, A 1691 Kern, F 229, 1240 Kerr, D 1529 Keski-Säntti, H 1890 Kesty, NC 1762 Kettner, E 1071 Key, TJ 6 Khambata-Ford, S 488 Khan, G 1362 Khan, MN 1151 Khan, OA 750, 1822 Kharbili, MEI 155 Khatri, G 892 Khaw, K-T 1493 Kheifets, L 228 Kikkawa, N 376 Killian, A 1517 Kim, CG 1126 Kim, EJ 1730 Kim, H-S 605 Kim, HL 643 Kim, IA 1730 Kim, JH 1730 Kim, K-p 605 Kim, KH 559 Kim, M 290 Kim, NK 1126 Kim, S-W 1730 Kim, TW 605 Kim, WH 166 Kim, YH 166 Kim, YII 1027 Kim, YJ 1730 Kim, YW 1126 Kim-Sing, C 1384 Kimura, H 700 Kimura, S 91 Kinlen, L 12 Kiriakidis, S 1151 Kirichek, O 1067 Kita, Y 714 Kleibeuker, JH 37 Kline, K 101 Klomp, M 1020 Kloor, M 1334 Knight, JA 1906 Knijn, N 1020 Knoll, C 469 Knösel, T 1013 Knowles, MA 75, 1135 Knudsen, AB 1779 Ko, JMY 841 Ko, YS 166 Koffijberg, H 1325 Kohno, K 1882 Kollarova, H 1797 Kondo, C 856 Kondo, M 819 Kondo, S 1160 Kong, S-Y 1126 Konishi, N 700 Konopleva, M 91 Konstantopoulou, I 1356 Kooi, N 1278 Kornfeld, J-W 138 Koschel, A 1426 Kostourou, V 83 Koszik, F 469 Kote-Jarai, Z 175 Kotsori, A 1675 Koyama, S 1349 Krautkrämer, E 1334 Krishnan, K 524 Kroll, ME 1227 Kruitwagen, RF 221 Kruse, AJ 221 Kruyt, FAE 1185 Kuan, R 1246 Kuhns, MA 957 Kullar, PJ 1810 Kumar, S 1038 Kuntz, KM 1779 Kurahara, H 714 Kuroda, J 91 Kurokawa, Y 707 Kuroshima, T 819 Kurosu, T 819 Kuten, A 1649 Kuwata, K 1594 Kwan-Lim, GE 578

La Vecchia, C 1207 LaBaer, J 1896 Labianca, R 1786 Laccetti, P 1716 Lacevic, M 1828 Lacey, M 1221 Lachuer, J 155 Lackner, A 1303 Lagiou, P 1493 Lagoudaki, E 316 Lagrasta, CA 1372, 1929 Lai, PBS 1000 Lajer, CB 830 Lalloo, F 1356 Lam, MYY 1000 Lamartine, J 155 Lamont, FR 75 Lane, JA 875 Langer, CJ 68 Langers, AM 37 Langner, C 1641 Languino, LR 629 Larkin, JMG 399, 407, 750 Lasa, A 1356 Lash, TL 188, 1804 Lattanzi, A 248 Lau, PY 369 Lau, WKO 941 Laughlin, A 756 Laughton, CA 653 Launay, O 1670 Laurent, D 1686 Law, M 886 Lawler, MP 281 Lawrence, G 1680 Lawrence, YR 1365 Le Chevalier, F 1670 Le Naour, F 155 Leach, MO 578 Lebon, P 1670 Lee, AJ 1697 Lee, BL 166 Lee, HE 1730 Lee, J-L 605 Lee, JE 559 Lee, JH 1126 Lee, JK 1027 Lee, JY 1027, 1126 Lee, KH 1027 Lee, KT 1027 Lee, PWK 290 Lee, SS 605 Lee, VHM 1000 Lee, Y-f 369 Leek, R 1168 Legrand, E 496 Legres, L 1739 Lehmann-Che, J 1739 Leighl, NB 413 Leonardi, F 1372, 1929 Leong, HS 971 Leong, T 265 Lester, N 1529 Leunen, K 863 Leung, HY 664 Levine, L 353 Lew, JQ 537 Lewandowski, P 1055 Li, D 68 Li, H 1762 Li, H-L 1511 Li, J 1523 Li, J-L 841 Li, L 19 Li, PJ 841 Li, Z 1882 Lian, JB 629 Liao, D 1410, 1931 Liao, S-Y 353 Liao, YH 982 Liberski, PP 968 Ligtenberg, MJ 1356 Liljegren, A 1356 Lim, GH 871 Lim, H-S 605 Lim, L 741 Limpaiboon, T 1313 Lin, J 464 Lin, S-R 1178 Lindman, H 899 Lindor, NM 1356 Lindström, MS 1747 Linecker, A 1641 Linton, K 719 Lippi, G 1234 Little, J 255 Liu, J 892, 1241 Liu, J-F 175 Liu, P 1564 Liu, R 369 Lloyd, A 1049 Lococo, F 1877 Lofts, F 407 Logie, C 554 Loken, SD 290 Loman, N 1356 Lomnytska, MI 110 Lonardi, S 1418 Long, GV 392 Longtine, J 1896 Löning, T 138 Lopes, C 198 Lophatananon, A 175 Lorch, J 1896 Loric, S 1056 Lorigan, P 750 LoRusso, PM 1862 Losekoot, N 1185 Lou, Z 19 Loulergue, P 1670 Loupakis, F 1262, 1786 Lowenthal, RM 228 Lu, C-Y 1178 Lu, J 1270 Lu, Q 781 Lubin, J 181 Lucas, R 469 Luckyanov, N 181 Ludeman, L 1611 Luk, LY 1000 Lund, E 1493 Lundin, J 1890 Lunet, N 198 Lung, ML 841 Luo, Y 1410, 1931 Luppi, G 1079 Lyerly, HK 1575 Lyman, R 1221 Lynch, HT 1384 Lyons, D 915

Ma, BBY 1000 Ma, X-J 1762 Ma, YT 1500 MacArthur, S 361 MacDonagh, B 281 Machesky, L 664 Macleod, U 1697 Maddala, T 488 Maders, F 719 Madhusudan, S 653 Madsen, M 520 Maginn, EN 281 Maishi, N 819 Maitland, N 673 Majid, S 892 Mäkinen, LK 1890 Makino, T 707 Malavasi, N 1079 Malmström, P-O 899 Maloney, S 1611 Mandolesi, A 1786 Manganelli, A 613 Mangia, A 769 Mania, E 248 Manjer, J 1493 Manoukian, S 1356 Mantle, M 741 Margison, GP 750 Markowitz, D 1410, 1931 Marom, O 1649 Marquez, R 664 Marreaud, S 1544 Marrelli, D 1770 Marsili, LM 248 Martens, JHA 554 Martin, FL 790 Martin, P-M 332, 1364 Martin, RM 875 Martinelli, E 427 Martin-Hirsch, PL 790 Masi, G 1262 Masur, K 345 Masuzawa, T 707 Masyakin, VB 181 Matera, A 265 Mates, D 1797 Mathoulin-Pelissier, S 1544 Mathus-Vliegen, EM 37 Matsuda, R 376 Matsumoto, M 714 Matsumoto, Y 1349, 1724 Matsumura, Y 593 Matsuura, N 707 Matte, I 989 Matveev, V 1797 Maughan, T 1529 Mauguen, A 1144 Mauland, KK 921 Maurer, D 469 Mauro, DJ 488 Mavroudis, D 316 Maxwell, PH 1151 Maxwell, PJ 480 May, A 1493 May, MT 1755 Mazouni, C 332, 1364 McCall, P 1920 McCarthy, K 1611 McCarthy, NS 338 McClean, M 1896 McConnell, RJ 181 McCormack, L 1602 McCracken, SRC 664 McElligott, AM 281 McFaul, S 620 McGown, G 750 McGuffog, L 1356 McGuinness, DH 1920 McKendick, J 265 McLachlan, J 43 McLaughlin, JR 1906 McLean, C 524 McLennan, J 1384 McMillan, DC 726 Meder, S 138 Medhaoui, D 1517 Medioni, J 1670 Medley, L 1067 Meersma, GJ 1278 Mehta, M 1365 Meijer, A 1278 Meijer, CJLM 685 Meijer, J 227 Meijer, JWR 1020 Mekenkamp, LJM 1020 Melisko, M 1828 Menuel, C 1854 Mesher, D 915 Mesia, R 1691 Metcalfe, K 1384 Meyer, C 1246 Mezei, G 228 Miceli, R 1256 Michael, M 265 Michalski, JM 1365 Michikawa, C 850 Michlmayr, A 543 Middleton, MR 750, 1822 Miida, T 91 Miles, T 1836 Miller, A 1828 Miller, CJ 971 Miller, CR 545 Mills, R 620 Milne, RL 1356 Milner, AD 265 Minenko, VF 181 Minton, SE 1828 Mints, M 110 Mitchell, E 1697 Mithal, N 620 Miyamoto, Y 1349 Miyata, H 707 Mizota, A 856 Moasser, M 1828 Mohammed, MZ 653 Mole, DJ 1007 Molina-Garrido, MJ 224 Møller, S 419 Möller-Levet, C 971 Molloy, TJ 1913 Montella, M 1207 Moons, KGM 1325 Moore, LE 1797 Moore, N 413 Morel, AN 1067 Moreno, VR 735, 1519 Morgan, C 1755 Morgan, DAL 1393 Morgillo, F 427 Mori, M 707 Morland, R 664 Morrison, DS 726, 1791 Morrison, EE 1602 Moser, R 1529 Moskal, A 1493 Moss, SM 571, 910 Mouw, T 1493 Moyret-Lalle, C 387 Mracek, T 441 Mrkonjic, M 1906 Muir, K 175, 193 Mukherjee, R 1920 Mulder, NH 1193 Mulè, A 1877 Müller-Spahn, C 948 Munneke, BM 488 Munro, AJ 60 Munster, PN 1828 Murata, H 464 Murdoch, J 1836 Muro, K 856 Murphy, MFG 1227 Murray, MJ 746 Murray, PG 1500 Musselman, JRB 214

Nadesan, P 1452 Nagasaka, K 1349 Nagengast, FM 37 Nagtegaal, ID 1020 Nakada, M 798 Nakagawa, H 300 Nakagawa, K 1349, 1594 Nakagawa, M 376, 808 Nakagawa, S 1349 Nakajima, K 707 Nakamura, Y 300 Nakano, R 1882 Nallaswamy, V 1836 Nam, B-H 1126 Nam, J-M 433 Nam, KT 33 Nam, SJ 559 Nam, SY 166 Nambu, E 798 Napolitano, V 427 Narod, SA 1384 Naska, A 1493 Nathan, P 741 Nathanson, KL 1356 Natsugoe, S 714 Nauntofte, B 830 Navarro, C 1493 Navratilova, M 1797 Neal, DE 875, 1656 Negri, E 1207 Negri, FV 1372, 1929 Neoptolemos, JP 1440 Neri, D 1106 Nesland, JM 1434 Nevanlinna, H 1356 Newcomb, PA 763 Newell, DR 1869 Newton, GE 971 Newton, L 1836 Ng, CKY 361 Ng, LK 460 Ng, SSM 893, 1000 Ngan, S 265 Niault, T 229, 1240 Nichols, PW 1482 Nielsen, FC 830 Nieuwenhuis, MH 37, 1237 Niggemann, B 345 Nishimura, Y 300 Nishio, K 1594 Nishiya, T 593 Nishiyama, K 376, 808 Nistér, M 1747 Nixon, C 664 Nizzoli, R 1372, 1929 Njølstad, TS 921 Noble, TW 1551 Nohata, N 808 Nomura, M 856 Nong, RY 1619 Nonomura, K 241 Norat, T 1493 Nordenskjöld, B 899, 1762 Nørgaard, M 1202 Norrild, B 830 Norvell, J 1628 Novello, S 68 Novotny-Diermayr, V 756 Nuttall, RK 664 Nybo Andersen, A-M 520

Oakley, C 407 Oberg, I 1810 O’Brien, L 175 O’Brien, M 1675 O’Brien, N 620 O’Connor, JPB 719 O’Connor, M 419 Oda, K 1349 Odicino, FE 1418 O’Dwyer, PJ 1862 Oehler, R 543 Offman, J 871 Ogawa, H 505 Ogink, J 227 Ognjanovic, S 532 O’Grady, A 480 Ohga, N 819 Ohyama, Y 850 Okada, N 850 Okamoto, I 1594 Okamoto, W 1594 O’Kane, P 181 Okera, M 407 Oksuzyan, S 228 Olabi, B 1007 Oladipo, O 480 Olesen, F 934, 1249, 1930 Oliphant, R 1791 Oliveira-Cunha, M 514 Oliver, AW 324 Oliver, CT 1356 Oliver, T 620 Olmos, D 1 Olofsson, T 1747 Olopade, OI 1384 Olsen, A 1493 Olsson, A 110 Olver, B 1049 O’Neill, C 1007 Ong, K-r 1356 Orange, C 1920 Orditura, M 427 O’Reilly, DSJ 726 Orlandi, P 1262 Orrego, A 1747 Orsini, N 1196 Osler, M 520 Osorio, A 1356 Österlund, P 599 Osuji, N 407 O’Sullivan, E 1680 Oswal, A 1810 O’Toole, K 1869 O’Toole, L 1529 Otsuka, N 241 Otten, JDM 910 Ottley, CJ 593 Ouafik, L 332 Ouali, M 1544 Oudard, S 1670 Overvad, K 1493 Øyan, AM 921

Paap, E 910 Pagano, JS 1160 Page, E 175 Page, K 1342 Påhlman, L 1619 Pai, SI 1896 Pala, V 1493 Palli, D 1493 Pallone, F 1770 Palumbo, A 1106 Panico, S 1493 Pantel, K 138 Papadaki, C 316 Papadopoulos, P 1675 Papierz, W 968 Paradiso, A 769 Parfrey, PS 1906 Park, JW 166 Park, SR 1126 Park, SY 1730 Park, Y 537 Park, Y-I 1126 Park, YH 559 Parker, T 175 Parmar, M 1529 Parton, M 1675 Pascucci, A 613 Pashayan, N 1656 Passarelli, MN 763 Patel, II 790 Patel, PM 653 Paterson, I 1319 Patil, M 1168, 1822 Paul, AB 1135 Pauwels, P 1472 Pawade, J 1755 Payne, MJ 1822 Paz-Ares, LG 68 Pearmain, P 1505 Pearson, DG 593 Pecorelli, S 1418 Pedersen, AF 1249 Pedersen, L 1202 Peeters, DJE 1472 Peeters, M 1472, 1848 Peeters, PH 1493 Peissel, B 1356 Peleteiro, B 198 Pellegrini, A 248 Penel, N 1544 Peng, G 1649 Peock, S 1356 Pepper, SD 971 Pereira, SP 587 Perks, CM 1587 Persad, RA 1587 Persson, C 1511 Peterlongo, P 1356 Peters, FT 37 Peters, GJ 437, 1185 Petersen, I 1013 Petitpain, N 1238 Peto, J 389 Peto, R 1057 Petrioli, R 613 Pettorelli, E 1079 Pezzella, F 1877 Pflueger, M 469 Pharoah, P 564, 1656 Pharoah, PD 693 Philip, PS 1362 Phillips, K-A 903 Phillips, L 1755 Phillips, M 1246 Piaskowski, S 968 Picard, M 1691 Piché, A 989 Pichler, M 1641 Pickering, L 407 Pigozzo, J 1816 Pinard, M 989 Pinto, C 427 Pirson, S 948 Pita, G 1356 Plassa, L-F 1739 Plummer, R 593 Pocock, R 175 Pogoda, JM 1482 Pointecouteau, T 155 Pointon, LJ 578 Polesel, J 1207 Pollak, MN 68 Pollett, A 1906 Polterauer, S 223 Polyanskaya, ON 181 Ponchietti, R 613 Pond, GR 1711 Ponnusamy, MP 1038 Pontén, F 1619 Poon, R 1452 Popat, S 407 Porcelli, L 769 Porta, C 1256 Posner, M 1896 Potter, JD 763 Potts, A 1221 Potts, V 1529 Poussa, T 599 Powers, J 756 Powles, T 620, 741 Preiss, L 433 Preston, G 1116 Preston-Martin, S 1482 Pretto, F 1106 Price, N 1459 Price, SJ 1810 Pridgeon, SW 1869 Pring, M 1459 Proby, CM 1459 Procopio, G 1256 Proctor, MJ 726 Pronk, GJ 272 Protheroe, A 620 Protheroe, AS 1822 Prové, A 1472 Provenzano, E 693 Pucciarelli, S 587 Puisieux, A 387 Punt, CJA 1020 Purcell, C 480 Pyko, IV 798

Qi, RZ 841 Qiu, H 208 Qu, M 1747 Quatrale, AE 769

Rachagani, S 1038 Radford, J 719 Radice, P 1356 Raeder, MB 921 Ragnoli, M 1418 Ragoussis, J 1168 Raha, P 1828 Rahman, AA 175 Rahman, S 43 Ramachandran, A 1168 Ramachandran, V 1270 Ramage, JK 587 Ramsay, AK 664 Ramsey-Goldman, R 1478 Ramus, SJ 903 Rancourt, C 989 Rancourt, DE 290 Randolph, SS 1862 Ranson, M 719 Raskett, CM 629 Rasmussen, H 1434 Rau, T 138 Ravaggi, A 1418 Rawson, JB 1906 Raymond, E 272 Razak, ARA 756 Rebbeck, T 1356 Reed, MWR 1551 Reed, N 1067 Reeves, G 1487 Regan, D 886 Reinthaller, A 223 Reisfeld, RA 1410, 1931 Rennert, G 1519 Rennie, IG 1098 Reuschenbach, M 1334 Revaud, D 1056 Rhee, J 1126 Rhee, JC 1027 Riboli, E 1493 Ribom, D 1747 Ricci, S 1256 Rich, AM 460 Richardson, J 1602 Richter, GHS 948 Ridolfi, L 1256, 1816 Ridolfi, R 1816 Riemer, AB 1896 Riemersma, S 1020 Rieske, P 968 Riethdorf, L 138 Riethdorf, S 138 Riis, AH 188, 1804 Rischin, D 265 Ristimäki, A 1890 Rizk, NP 1840 Robertson, C 1221 Robertson, D 188, 1804 Robinson, BA 51 Robinson, M 1319, 1869 Robinson, SP 83 Robinson, V 1007 Robison, LL 532 Röcken, C 1426 Rodgarkia-Dara, C 1303 Rodgers, WH 353 Rodrigues, G 673 Rodríguez, L 1493 Roesler, M 532 Rohrmann, S 1493 Romaguera, D 1493 Romain, S 332 Romani, C 1418 Romanini, A 1816 Romano, N 433 Romanov, GN 181 Ron, E 181 Roos, E 227 Ropert, S 1670 Rosales, R 1434 Rösel, S 1071 Rosell, J 899 Rosen, B 1384 Ross, JA 441, 532 Ross, RK 1482 Rossing, M 830 Rothman, N 1511, 1797 Routledge, P 927 Roviello, F 1770 Rowinsky, EK 488 Rozek, LS 735 Rozendaal, L 685 Rozhko, AV 181 Ruangpratheep, C 1342 Rugo, H 1828 Rumsby, M 673 Ruol, A 427 Russell, I 255 Rydén, M 441 Ryu, HS 1730 Ryu, KW 1126 Ryu, M-H 605

Sablin, M-P 272 Sacco, C 1256 Sadetzki, S 1049 Saeed, S 554 Saida, T 464 Saini, S 892 Sakai, K 1594 Sakaizawa, K 464 Sakata, K-i 1724 Sala, E 361 Salunga, R 1762 Salvatore, L 1262 Salvesen, HB 921 Samonigg, H 1641 Samuel, L 1697 Samuel, S 1270 Sánchez, M-J 1493 Sanders, BG 101 Sandstad, B 1434 Sani, C 248 Sany, O 1505 Sarker, SJ 620 Sasaguri, Y 1882 Sato, H 850 Sawyer, E 175 Scala-Bertola, J 1238 Scarabelli, L 1079 Scartozzi, M 1786 Schaake, E 1325 Schaller, G 1611 Schatzkin, A 537 Schellens, JHM 1913 Schiboni, ML 248 Schirripa, M 1262 Schmitt, J 673 Schoffski, P 1544 Schouten, WR 37 Schuett, W 469 Schulte-Hermann, R 1303 Schüz, J 228 Schwager, K 1106 Schwartz, GK 1862 Sebastian, S 769 Seguí, N 735 Seir, K 1303 Seki, N 376, 808 Selby, P 1529 Sellars, SJ 571 Semba, S 146 Senapati, S 1038 Senju, S 300 Sereno, E 248 Serova, M 272 Severi, G 524 Sexton, JZ 1575 Seymour, GJ 460 Seywright, M 664, 1920 Sezer, O 587 Sgroi, DC 1762 Shah, MA 1840 Shah, S 653 Shahryari, V 308, 892 Shaik, MN 1862 Shak, S 488 Sham, PC 369 Shamash, J 620 Shan, H 505 Sharma, A 1342 Sharp, DM 578 Sharp, L 255 Sharrard, M 673 Shaw, JA 1342 Sheldon, H 1168 Sheng, Q 1241 Sherriff, J 927 Sherrill, B 1848 Shi, Q 1410, 1931 Shibata, N 856 Shiirevnyamba, A 505 Shimada, K 700 Shimajiri, S 1882 Shimizu, C 241 Shin, J-G 605 Shindoh, M 819 Shinohara, N 241 Shirane, A 1349 Shitara, K 856 Shnyder, SD 75 Shoji, K 1349 Shrikant, PA 643 Shu, X-O 1511 Sibbering, DM 1551 Siemerink, EJM 1193 Sieruta, M 968 Siesling, S 1193 Sikand, KA 971 Sileni, VC 427 Sileri, P 1770 Silini, EM 1372, 1929 Sim, MY 941 Sim I, HG 941 Simone, GM 769 Singh, K 308 Sinha, A 1236 Sini, P 769 Sinilnikova, OM 1356 Sinka, K 1221 Sirab, N 1056 Siriwardena, AK 514 Sisley, K 1098 Siu, LL 756 Skeie, G 1493 Skoog, L 1762 Slamova, A 1797 Slangen, BF 221 Sleijfer, S 1544 Slimani, N 1493 Sloan, P 1319 Sludden, J 593 Smart, L 255 Smith, I 1675 Smith, LD 903 Smith, LM 1038 Smith, MT 635 Smith, RA 1440 Smits, A 1747 Sohail, M 1755 Soliman, H 1739 Soltermann, A 1106 Someya, M 1724 Sommerer, C 1334 Sone, K 1349 Soofi, M 664 Sørensen, HT 188, 1202, 1804 Souglakos, J 316 Souhami, L 1365 Southey, MC 903 Soveri, L-M 599 Spaëth, D 1238 Specht, L 830 Spector, LG 214, 532 Sperati, A 248 Spirtos, NM 353 Spitalieri, G 1686 Spreafico, C 1686 Spurdle, AB 1356 Sriraksa, R 1313 Stål, O 899, 1762 Stanbridge, EJ 353, 841 Stanson, J 1641 Starling, N 1675 Stathopoulos, E 316 Stawski, R 968 Stayner, L-A 756 Stead, M 1529 Stecker, K 1426 Steele, RJC 60 Stefansson, IM 921 Stein, A 886 Stein, GS 629 Stemmler, HJ 1071 Stenmark-Askmalm, M 1356 Stephens, NA 441 Stevens, KN 735 Stevenson, M 480 Steverlynck, C 496 Stewart, D 1529 Stewart, PS 1797 Stewart-Brown, S 175 Stiller, CA 1227 Stockton, DL 60 Stoczynska-Fidelus, E 968 Stone, J 750 Storm, H 178 Stotter, A 1551 Stower, M 673 Stram, DO 1482 Stringfellow, HF 790 Strong, VE 1840 Stuart, N 1535 Stuifbergen, WN 37 Stunnenberg, HG 554 Subjeck, JR 643 Sugimoto, T 376 Suh, SO 892 Suissa, S 1558 Sullivan-Gunn, M 1055 Sun, P 1384 Sundar, S 1505, 1611 Sung, JJY 893 Sung, L 1377 Susumu, N 353 Swanson, J 228 Syed, BM 1393 Sykes, H 407 Szarewski, A 915 Szeszenia-Dabrowska, N 1797 Szybka, M 968

Tabatabai, ZL 308 Tabe, Y 91 Taillibert, S 1854 Takahari, D 856 Takahashi, M 241 Takahashi, T 505 Takata, M 464 Takeda, M 1594 Taketani, Y 1349 Takezawa, K 1594 Takiguchi, S 707 Talamini, R 1207 Talbot, DC 1067, 1822 Talwar, D 726 Tamagnini, I 1372, 1929 Tan, LT 361 Tanaka, Y 892 Tang, J 1440 Tang, LH 1840 Tanikawa, M 1349 Tappenden, N 1680 Targher, G 1234 Tasaki, M 700 Tassi, RA 1418 Tatarano, S 376, 808 Tavani, A 1207 Tavaré, JM 1755 Tavecchio, M 629 Taylor, GA 1869 Taylor, M 1144, 1168, 1822 Taylor, MB 719 Taylor, SE 790 Tebar, M 1020 Teerenstra, S 1020 Tellez, JD 1628 Tempfer, C 223 Temple, J 361 Teng, L 798 Terrazzano, G 1716 Tesch, H 1071 Tesoriero, AA 903 Tessen, HW 1071 Teuffel, O 1377 Teulé, A 1356 Tewari, P 281 Thakker, RV 1067 Thériault, C 989 Therkildsen, MH 830 Thiel, U 948 Thijssen, VL 1185 Thomas, DC 1482 Thomas, EC 1755 Thomas, R 265 Thomas, S 1828 Thomas, W 338 Thomas, ZI 1575 Thompson, D 578 Thomson, S 1810 Thomson, WM 460 Thorncroft, M 750 Thornton, A 255 Thurn, KT 1828 Tian, H 1628 Tilby, MJ 593 Timmerman, D 863 Tinat, J 1517 Tisch, U 1649 Tittarelli, A 228 Tiwary, R 101 Tjønneland, A 1493 To, S-h 369 Todeschini, P 1418 Toffalorio, F 1686 Toki, K 376 Tol, J 1020 Tomas, N 345 Tomita, Y 300 Tomlinson, DC 75 Tomlinson, IP 369 Tørring, ML 934, 1930 Totsuka, Y 819 Tournay, E 1144 Toyota, M 1724 Tran, T 1334 Tranter, N 620 Trayhurn, P 441 Treasure, T 1085 Trevisan, J 790 Trichopoulos, D 1493 Trichopoulou, A 1493 Trigo, JM 1691 Troiani, T 427 Trovik, J 921 Tryfonidis, K 316 Trypaki, M 316 Tsai, H-L 1178 Tsai, MS 982 Tsaroucha, E 316 Tse, LA 208 Tseng, D 1805 Tsujikawa, K 700 Tsukioka, S 1594 Tsunoda, T 300 Tsuruga, T 1349 Tudur-Smith, C 1440 Tumino, R 1493 Tung, N 1384 Turner, EL 875 Turpin, E 1739 Tylko-Hill, K 1836

Uchida, Y 376 Uchikado, Y 714 Uchiyama, A 464 Uehara, H 505 Uen, Y-H 1178 Ueno, K 308 Ueno, S 714 Uenosono, Y 714 Underwood, MA 1920 Ura, T 856 Urbanski, SJ 290 Utsunomiya, S 856 Uzawa, N 850 Uzoh, CC 1587

Valencik, M 1628 Valenti, RM 433 Valentine, HR 971 Valle, L 735, 1519 Vallée, J-N 1854 van Asperen, CJ 1356 van Dalsen, AD 37 van Dam, P-J 1472 van Dam, PA 1472 van Dam, R 37 Van den Eynden, GG 1472 van der Aa, MA 1193 van der Bij, S 1325 van der Bijl, J 37 van der Bilt, ARM 1278 van der Vijgh, WJF 437 van der Zee, AGJ 1278 van Dooren, MF 1356 van Gemert, WG 37 Van Gorp, T 221, 863 van Kemenade, FJ 685 van Krieken, JHJM 1020 Van Laere, SJ 1472 Van Roozendaal, CE 1356 van Schoor, G 910 Vandenberghe, E 281 Vannier, JP 496 van’t Veer, LJ 1913 Varna, M 1739 Vasen, HFA 37, 1237 Vasilakis, C 1085 Vasquez-Medrano, DA 1805 Vasse, M 496 Vaughan, V 1055 Vecchione, L 427 Vecht, J 37 Vedsted, P 934, 1249, 1930 Verbeek, ALM 910 Vergote, I 863 Verhoef, S 1356 Vermeulen, PB 1472 Vermorken, JB 1691 Verrando, P 155 Verzoni, E 1256 Vetter, C 345 Vidaud, M 272 Vieth, M 1426 Vijayakrishnan, J 1049 Vimond, N 1144 Vincent, C 635 Vincent, EE 1755 Vinceti, M 228 Vineis, P 1493 Vink-Börger, ME 1020 Vinokurova, S 1334 Visioli, CB 248 Visser, O 685 Vitucci, M 545 Voest, EE 1913 Volkova, E 51 von Buchwald, C 830 von Deimling, A 1747 von Knebel Doeberitz, M 1334 von Ruesten, A 1493 Voss, MA 1611, 1836 Vreeswijk, M 1356 Vyjayanti, VN 653

Wada-Hiraike, O 1349 Waddell, T 1675 Waisfisz, Q 1356 Walker, AJ 193 Walker, J 915 Walker, JL 353 Walker, L 255, 1356 Walker, LG 578 Walker, RA 1342 Walkington, L 1665 Waller, J 915 Wallgren, A 899 Wallin, A 1196 Wallin, U 1619 Wallis, MG 1680 Walsh, W 756 Walter, K 1296 Wang, J 1848 Wang, J-Y 1178 Wang, K-Y 1882 Wang, L 19 Wang, LD 841 Wang, M 1365 Wang, W 1564 Wang, X-r 208 Wang, X-Y 643 Wang, Y 643 Ward, B 1342 Ward, T 719 Wareham, N 1493 Waring, K 1836 Warmington, S 927 Warwick, J 120 Watanabe, T 146 Watson, AJ 750 Watson, D 488 Watts, C 1810 Waugh, DJJ 480 Waugh, N 255 Webster, J 1665 Weerakkody, RA 1810 Wei, W 1611 Weller, D 1697 Wellman, S 750 Wells, JE 51 Wernli, KJ 763 West, CML 971, 1168, 1319 Wheeler, JL 390 Whiteside, TL 1641 Whitley, GStJ 83 Whynes, D 255 Wieckowski, E 1641 Wiedenmann, B 1426 Wierinckx, A 155 Wiesner, C 469 Wiezorek, J 1848 Wik, E 921 Wilde, DJ 1551 Wilkinson, N 1602 Wilkinson, RA 175 Williams, DC 281 Williams, KP 1575 Williams, L 1007 Williams, RM 746 Willis, JA 51 Wilner, KD 1862 Wilson, C 480 Wilson III, DM 653 Wilson, P 620 Wilson, R 1529 Wilson, RH 480, 593 Wilson, S 927 Winship, I 903 Winterbottom, L 1393 Wirfärt, E 1493 Wishart, GC 564 Wohlberg, M 138 Wolanczyk, M 968 Wolfgang, C 1296 Wolin, KY 882 Wolk, A 1196 Wong, CS 871 Wong, DWM 1393 Wong, EEM 903 Wong, J 1896 Wong, SCC 1000 Wong, VCL 841 Wong, Y-w 369 Wood, J 1459 Wood, NJ 790 Woodman, CBJ 1500 Woolley, C 255 Wu, CW 893 Wu, D-C 1178 Wu, WKK 893 Wu, Y 1882 Wullschleger, S 1116 Wunsch Filho, V 228 Wyke, S 1697

Xia, L 1270 Xiang, R 1410, 1931 Xu, B 1564 Xu, J 1401 Xu, L-A 488 Xu, Y-Z 1869

Yadegarfar, G 587 Yamada, S 1882 Yamaguchi, H 1594 Yamasaki, M 707 Yan, Y 882 Yang, G 1511 Yang, HL 982 Yang, J-H 559 Yang, L 1013 Yang, TYO 4 Yang, W-M 781 Yano, S 505 Yano, T 1349 Yatabe, Y 856 Yau, CC 369 Yau, T-k 369 Ye, W 1511 Yi, J 413 Yip, NC 1564 Yokdang, N 1628 Yokomine, K 300 Yokota, T 856 Yokozaki, H 146 Yoon, J 166 Yoshino, H 376, 808 Yoshizaki, T 1160 Young, JP 1906 Young, L 338 Young, LS 1500 Younghusband, HB 1906 Yousaf, N 1675 Yu, AX 664 Yu, IT-s 208 Yu, J 893 Yu, W 101 Yuen, C-h 369 Yuen, JSP 941 Yun, J 559 Yung, W-KA 1365

Zablotska, LB 181 Zachariae, R 419, 1249 Zafar, SY 390 Zaffaroni, D 1356 Zahra, MA 361 Zalcberg, JR 265 Zaman, MS 308, 892 Zaniboni, A 1786 Zänker, KS 345 Zanotti, L 1418 Zaridze, D 1797 Zeier, M 1334 Zeller, C 1313 Zhan, Q 1523 Zhang, G 781 Zhang, R-W 781 Zhao, S-G 798 Zhao, Z 1848 Zheng, W 1511 Zheng, Y 763 Zhou, D 24 Zhou, H 1410, 1931 Zhou, W 128 Zhou, Y 91 Zhu, J 756 Zilembo, N 1256 Zironi, S 1079 Zisterer, DM 281 Zöller, K 1013 Zorzi, F 1786 Zucchetto, A 1207 zur Nieden, NI 2901

